# On the Evaluation of Flow Properties Characterizing Blown Film Extrusion of Polyolefin Alternatives

**DOI:** 10.3390/polym17172353

**Published:** 2025-08-29

**Authors:** Petr Filip, Berenika Hausnerova, Dagmar Endlerova, Bernhard Möginger, Juliana Azevedo

**Affiliations:** 1 Institute of Hydrodynamics, The Czech Academy of Sciences, Pod Patankou 30, 160 00 Prague, Czech Republic; filip@ih.cas.cz; 2 Faculty of Technology, Tomas Bata University in Zlin, Vavreckova 275, 760 01 Zlin, Czech Republic; d_endlerova@utb.cz (D.E.); julianavcazevedo@gmail.com (J.A.); 3 Department of Natural Sciences, University of Applied Sciences Bonn-Rhein-Sieg, von Liebig Str. 20, 53359 Rheinbach, Germany; bernhard.moeginger@h-brs.de

**Keywords:** shear viscosity, elongational viscosity, sagging, molecular weight, blown film extrusion

## Abstract

The lower melt strength of biodegradable materials in comparison to low density polyethylenes raises serious issues regarding their processability via blown film molding. Thus, reliable rheological characterization is a viable option for assessing their efficient flow performance. The blends of poly (lactic acid) (PLA) and poly (butylene adipate-co-terephthalate) (PBAT) modified with four chain-extending cross-linkers (CECLs) undergo shearing during extrusion and are subjected to extensional deformation during the subsequent film blowing. The shear viscosity data obtained with a capillary rheometer corresponded well to the molecular weights obtained by gel permeation chromatography, while an evaluation of elongational viscosity using a Sentmanat Extensional Rheometer failed due to sample sagging during the process of temperature setting and an unacceptable deviation from the theoretically supposed exponential decrease of sample cross-sections. Therefore, the response of the PBAT/PLA blends to elongation was determined via changes in the duration of time intervals corresponding to the rupture of elongated samples. An increased consistency of the PBAT/PLA blends with CECL, as previously indicated by dynamic mechanical analysis, differential scanning calorimetry, and scanning electron microscopy, was evaluated in this way.

## 1. Introduction

During the 20th century, conventional packaging materials such as glass and wood were progressively replaced by plastics, which offered clear benefits including low density, resistance to moisture, and excellent flexibility. Within the scope of film-blowing technology for packaging applications, low-density poly (ethylene) has proven to be the most stable material [1], as it provides the distinct strain-hardening behavior essential for successful blown film extrusion.

In recent decades, however, the rapid growth in packaging demand has coincided with the accumulation of municipal solid waste that is both non-biodegradable and resistant to biotic (microbial activity) and abiotic processes (oxidation, photodegradation). These circumstances make the need to transition from petroleum-based polymers to biodegradable alternatives increasingly urgent. For blown film extrusion, which requires thin and flexible products, the polymers employed must display a combination of high ductility, the ability to withstand large shear and extensional deformations, and sufficient melt strength [2,3].

Poly (lactic acid) (PLA), a linear thermoplastic polyester, satisfies environmental requirements [4] and also offers transparency and thermal processability. Nevertheless, its suitability for blown film extrusion is compromised by intrinsic drawbacks such as poor toughness, insufficient melt strength, brittleness (*T*g at 57 °C, elongation at break 3.8%), and limited thermal stability [5,6,7,8,9,10,11,12,13,14,15,16,17,18].

To address these limitations while maintaining PLA’s environmental benefits, it has been blended with other biodegradable polymers, including poly (butylene adipate-co-terephthalate) (PBAT), polycaprolactone, chitosan, and poly (butylene succinate adipate). These blends aim to improve ductility and other mechanical characteristics to make blown film extrusion feasible [19,20,21,22,23,24,25,26,27,28,29].

PBAT, although derived from fossil feedstocks, degrades within weeks in the presence of enzymes [30]. Structurally, PBAT contains two types of co-monomers: rigid butylene terephthalate segments (from 1,4-butanediol and terephthalic acid) and flexible butylene adipate segments (from 1,4-butanediol and adipic acid) [31,32]. With a glass transition temperature of −30 °C, PBAT demonstrates properties that complement PLA, including room-temperature ductility, low elastic modulus, elongation at break exceeding 500% (versus 3.8% for PLA), and high flexibility and toughness [31,33].

The miscibility of PLA with PBAT is limited to very low PBAT concentrations (reported up to 2.5 wt% [34], or 5 wt% [35]). At higher PBAT contents, three sequential morphological regimes emerge [34]. When PBAT is present up to 20 wt%, spherical droplets are dispersed in a PLA matrix. Between 20 and 50 wt%, the dispersed phase elongates into fibrils. At PBAT levels of 50–70 wt%, a co-continuous morphology develops, while above 70 wt% the system reverses, with PLA droplets distributed in a PBAT matrix. In reciprocal arrangements, PLA droplets (PLA/PBAT 20/80 *w*/*w*) are smaller and more regular compared to PBAT droplets (PLA/PBAT 80/20 *w*/*w*) [35]. Despite the relatively small difference in solubility parameters between the two polymers, immiscibility has been demonstrated through DSC (distinct *T*g values), SEM (visible phase separation), and rheological tests (two relaxation processes in Cole–Cole plots) [36].

For effective blown film extrusion, higher PBAT contents are generally necessary. These compositions enhance elongation at break, toughness, and shear-thinning tendencies [35,37,38,39,40,41]. However, this improvement comes at the expense of barrier performance against oxygen and water vapor [41]. According to Chiu et al. [42], optimal tensile and impact strength values were obtained at 70 wt% PBAT with an irregular sea–island morphology. Similarly, Pietrosanto et al. [41] identified an 80/20 PBAT/PLA ratio as particularly suitable for frozen food packaging down to −25 °C, with elongation at break unaffected by temperature [39]. Su et al. [36] reported that films containing 10–30 wt% PLA maintained consistent thicknesses, whereas higher PLA loadings led to unstable blowing. Importantly, the blends retain compostability, and the addition of catalytic fillers (e.g., ZnO particles) can accelerate mass loss by 75% within 90 days [43].

Despite these enhancements, PLA/PBAT blends still do not fully satisfy the demands of blown film extrusion due to their intrinsic immiscibility and the resulting weak interfacial adhesion [44].

One approach to compatibilize the system is through the addition of nanofillers, which reduce interfacial tension by localizing at the phase boundary [45,46,47]. Various nanofillers have been investigated, including epoxy-functionalized graphene [48], cellulose nanocrystals [49,50,51], nanoclays [48,52,53], graphene [48,49,50,51,52,53,54], carbon nanotubes [54], and nanosilica [54,55]. Their presence influences blend morphology by balancing droplet breakup and coalescence, thereby tuning stiffness and toughness. The effectiveness of nanofillers strongly depends on their distribution and localization in a blend [56,57]. They also improve durability by reinforcing mechanical properties [58,59,60,61].

Alternatively, compatibilization can be achieved with reactive additives. Epoxy-terminated branched polymers [28,62] reduce the *T*g difference between PLA and PBAT through physical and chemical micro-cross-linking, thereby enhancing elongation at break and impact strength. Reactive blending strategies involving cross-linking agents and chain extenders further improve adhesion at the PLA/PBAT interface, increasing melt elasticity, viscosity, and overall compatibility regardless of the blend ratio [25,40,63]. These modifications raise tensile strength, toughness, and elongation at break [40,63,64,65]. In unmodified blends, fractures propagate mainly along PLA- and PBAT-rich interfaces [63].

Chain extension and cross-linking induce complex rheological responses, including strain hardening due to increased entanglements and covalent bonding between the polymers [40]. Chain extenders may also introduce long-chain branching [14,25,66,67,68,69]. While neat PLA does not exhibit strain hardening [14], and uncompatibilized PLA/PBAT blends lack it at low extension rates (<1 s^−1^) where disentanglement dominates [14,70], reactive compatibilization allows strain hardening to emerge without compromising biodegradability.

The present study extends earlier investigations [71,72,73] by analyzing the influence of four multifunctional chain-extending cross-linkers (CECL) on shear and the elongational flow behavior of a reference PBAT/PLA blend (M·VERA^®^ B5029) [74] processed by blown film extrusion. A careful evaluation of rheological properties under shear and extension provides a pathway to optimize blend design for film-blowing operations and ensure the required mechanical quality of the final films.

## 2. Experimental Setup

### 2.1. Materials and Blown Film Manufacturing

Four chain-extending cross-linkers (1 wt%) were compounded with the reference PBAT/PLA blend (REF) M·VERA^®^ B5029 [71] from BIO-FED, Cologne, a branch of AKRO-PLASTIC GmbH, Hamburg, Germany. REF is mainly used in packaging and agricultural applications and consists of a PBAT/PLA blend with a weight ratio of approximately 6:1 and 24 wt% of calcium carbonate particles (D50 = 1.2 µm, top cut 4 µm, density 2.7 g/cm^3^, bulk density 0.55 kg/L, irregularly shaped particles). The number-average molecular mass *M*_n_ is 44,000 g/mol, the weight-average molecular mass is *M*_w_ 83,000 g/mol, and the polydispersity index of the polymer PDI is 1.89 [73].

The following four chain-extending cross-linkers were employed in 1 wt% amounts:

V1— tris (2,4-di-tert-butylphenyl)phosphite, Songnox 1680 (Songwon Industrial Co, Ulsan, Republic of Korea) [75];
V2— 1,3-phenylenebisoxazoline, 1,3-PBO powder (Evonik, Essen, Germany) [76];
V3— aromatic polycarbodiimide, Stabaxol P110 (Lanxess, Cologne, Germany) [77];
V4— poly (4,4-dicyclohexylmethane carbodiimide), Carbodilite HMV-15CA (Nisshinbo, Tokyo, Japan) [78].

The concentration of 1 wt% is the maximum of the percentage that did not disturb the biodegradation process. All ingredients were evenly mixed using a Mixaco CM 150-D (Mixaco Maschi-nenbau, Neuenrade, Germany) and compounded using a twin-screw extruder (FEL 26 MTS, Feddem GmbH, Sinzig, Germany) with a length/diameter ratio of 26, a screw speed of 260 rpm, and an output rate of 20 kg/h. The films were manufactured using a blow molding machine LF-400 (Labtech Engineering Company, Samut Prakan, Thailand) with an extrusion temperature of 165 °C and a blow-up-ratio (BUR, relating bubble diameter to die diameter) of 2.5:1 for 100 µm thick films.

CECLs affect the number-average molecular mass *M*_n_ (g/mol), weight-average molecular mass *M*_w_ (g/mol), and polydispersity index (PDI) of the REF blend (44,000, 83,000 and 1.89) in the following ways: the values slightly decreased for V1 to 42,000, 81,000, and 1.92, they increased for V2 to 46,000, 92,000, and 1.98, and they increased considerably for V3 (75,000, 149,000, and 1.99) as well as for V4 (72,000, 141,000, and 1.95) [73].

Consequently, the CECLs influence the melt flow ratio, and thus the melt viscosities of the compounds [37]. For the given blowing condition, this may affect film thicknesses and draw ratios (DR = width of die gap/(film thickness × BUR)). The film thicknesses listed in Table 1 exhibit relatively small standard deviations because they were determined on stripes before compost storage from a relatively short section of blown films. A device for in-line monitoring of the thickness was not available.

### 2.2. Methods

A capillary rheometer (RHEOGRAPH 50, Goettfert, Germany) equipped with a flat die (180° entrance angle) with the length-to-diameter (*L*/*D*) ratio (20/1) was used to evaluate the shear viscosities of REF and V1 to V4 blends at temperatures ranging from 140 to 160 °C. The apparent shear stresses were determined from the measured pressure drops and the length/diameter ratio of the capillary die. The shear rate range was set from 200 to 2000 s^−1^, which corresponds to the shear rate range relevant to film-blown extrusion. Finally, the apparent viscosity was calculated as the ratio of apparent shear stress to apparent shear rate.

**Figure 1:**
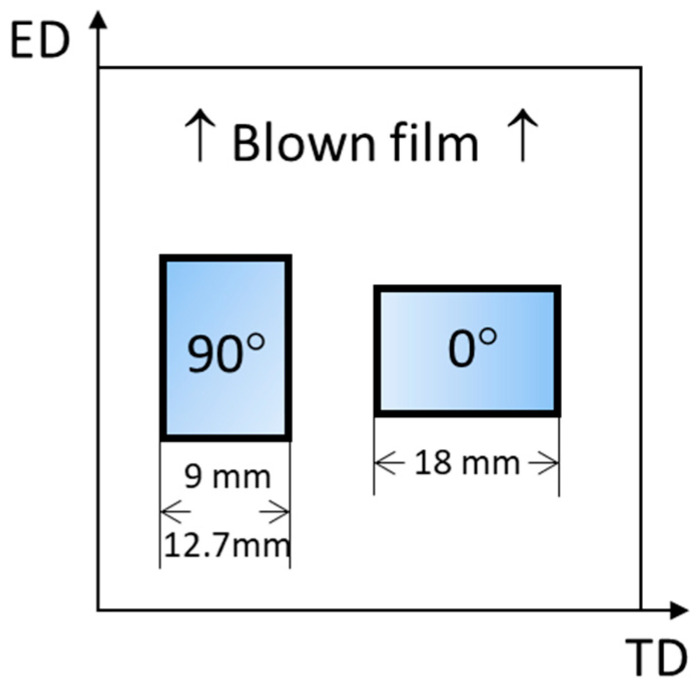
Sample taking and notation (extrusion direction—ED, transverse direction—TD).

A Sentmanat Extensional Rheometer (SER) Universal Testing Platform (model SER-HV-P01, Anton Paar, Graz, Austria) was used with an MCR501 (Anton Paar, Graz, Austria) rotational rheometer host system equipped with the convection heated measuring chamber CTD 450. Four extensional rates 0.0316, 0.1, 0.316, and 1 s^−1^ were adjusted via the rotational speed of the counter-rotating drums at temperatures ranging from 135 to 170 °C (given by the melting temperatures of PBAT and PLA, respectively). Measurements were performed at least in triplicate. Rectangular samples with a length of 18 mm (out of which 12.72 mm is active, i.e., stretched between both counter-rotating drums) and widths of 9 and 12.7 mm were taken from the blown films (with a thickness of approximately 0.1 mm, Table 1), both in the extrusion direction (ED, 90°) and in the transverse direction (TD, 0°) (see Figure 1). Two widths were chosen to verify the homogeneity of the blown films. Prior to testing, the films were conditioned for 24 h at 23 °C/50% relative humidity.

**Figure 2:**
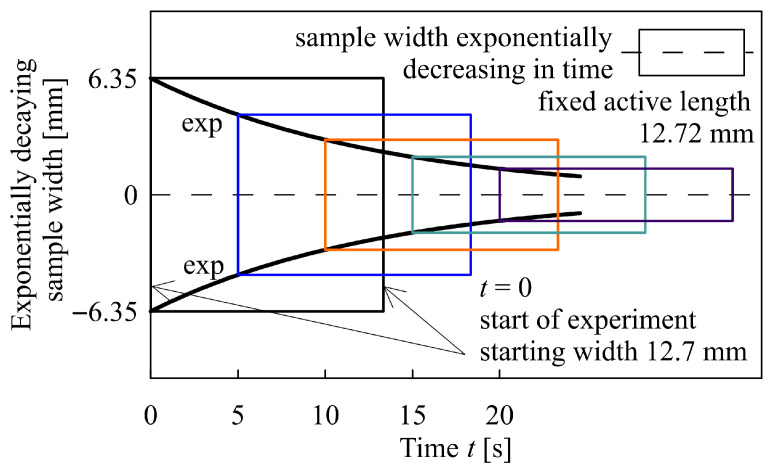
Consecutive change of a rectangular sample (time decay of its width with a fixed active length)—a narrowing rectangular area. Two initial sample widths were applied: 12.7 mm and 9 mm.

It should be stressed that the measurement of elongational viscosity does not extend to trivial operations. The ingenious devices proposed by Meissner [79] and Münstedt [80] in the 1980s are no longer in use. At present, the field is dominated by the Sentmanat Extensional Rheometer Universal Testing Platform (SER). The big advantage of this apparatus is its small size, which enables its practical use in any standard rotational rheometer. Easy manipulation makes it more comfortable to use. However, this tempts one to apply SER for polymer melts, which are not in compliance with the SER requirements formulated in Sentmanat [81]. For instance, in this case, one of the requirements that is not fulfilled (apart from the others) is the a priori exponential time decay of the sample’s upper and lower edges, see Figure 2. A failure of this assumption disables a determination of elongational viscosity—its numerical value. However, this device can be used for a characterization of the extension properties through the times corresponding to the rupture of the elongated samples.

**Figure 3:**
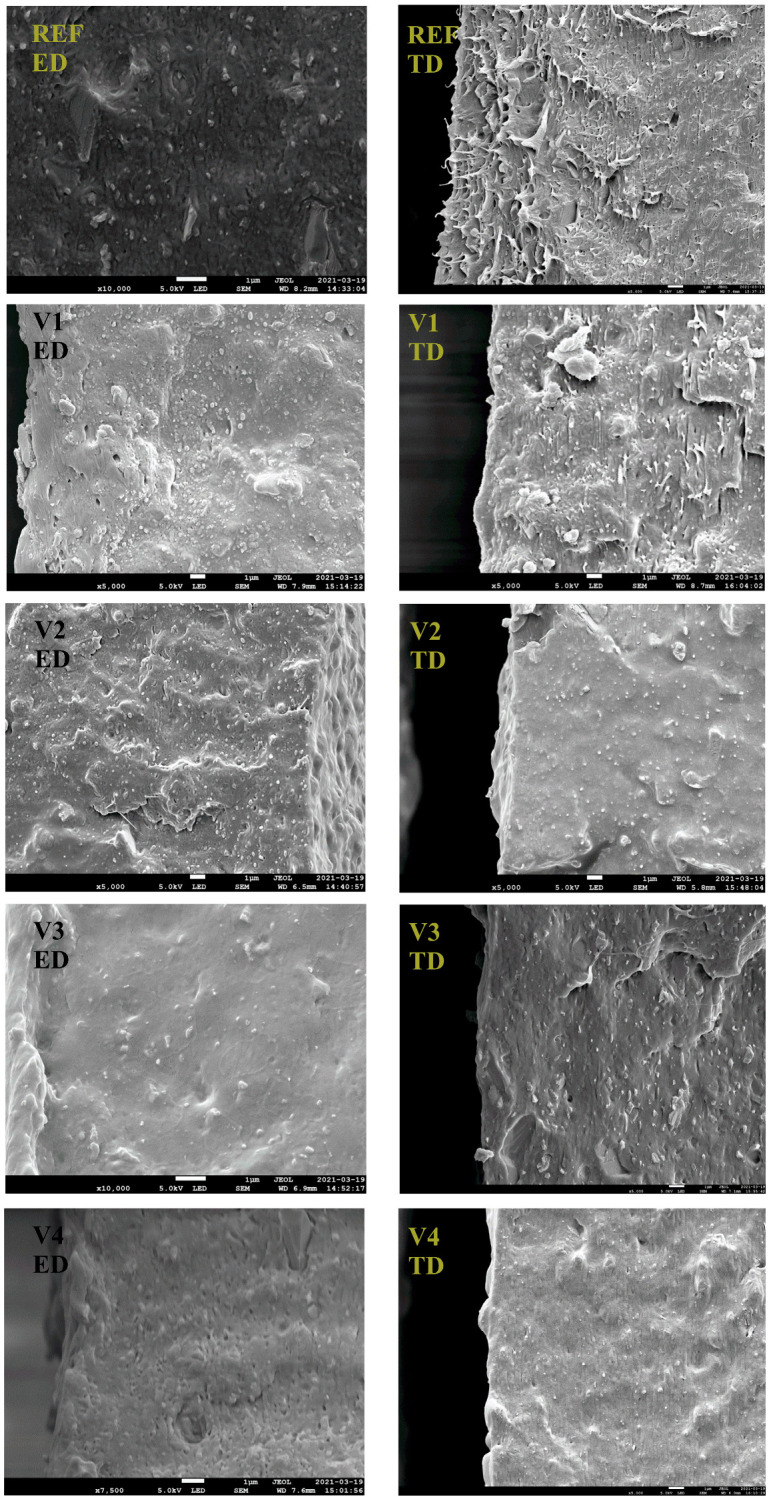
The SEM pictures of REF and V1 to V4. The indicated scale unit represents 1 μm.

A field-emission Scanning Electron Microscope SEM (JSM-7200F, Jeol, Tokyo, Japan) was used to investigate the morphology of the fracture surfaces of REF, and V1 to V4 with an acceleration voltage of 5.0 kV and amplifications of 3000 and 10,000, as seen in Figure 3. The films were cryo-fractured both in ED and TD to document morphology anisotropy.

The morphologies of REF, and V1 to V4 in ED and TD differ with respect to the sizes and shapes of the dispersed PLA phase, as shown in Figure 3. REF is characterized by an inhomogeneous dispersion of PLA in the PBAT matrix generating a ’sea–islands’ structure, resulting in relatively poor mechanical properties due to weak interfacial adhesion. The consequence is that the PBAT matrix experiences most of the deformation happening in a tough manner with pronounced fibrillations on the TD fracture surface.

On the ED fracture surfaces, V1 to V4 exhibit a morphology with the dispersed PLA phase having a spherical shape, whereas the morphology on the TD fracture surfaces differs due to the chosen chain-extending cross-linker. V1 and V2 exhibit similar morphologies and failure behavior but with less fibrillation on the TD fracture surfaces. Furthermore, the PLA droplets on the fracture surfaces have a fibrillar shape in the TD, with corresponding deformational effects. On the TD fracture surfaces, V3 exhibits droplets of spherical and ellipsoidal shapes, whereas V4 exhibits droplets of spherical and fibrillar shapes. A detailed SEM characterization is given in [72]. The structural differences shown in Figure 3 indicate that V4 is the most promising modification for blown film extrusion.

## 3. Results

**Figure 4:**
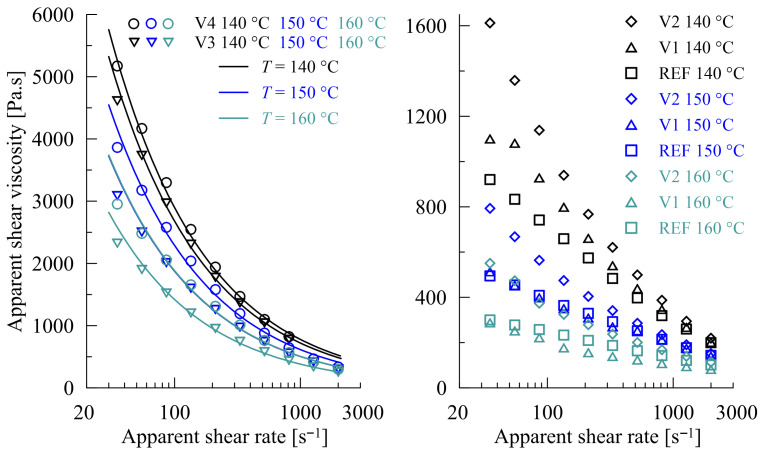
Effect of CECLs on shear deformation of REF and modified (V1 to V4) PBAT/PLA blends (arranged with respect to the samples).

**Figure 5:**
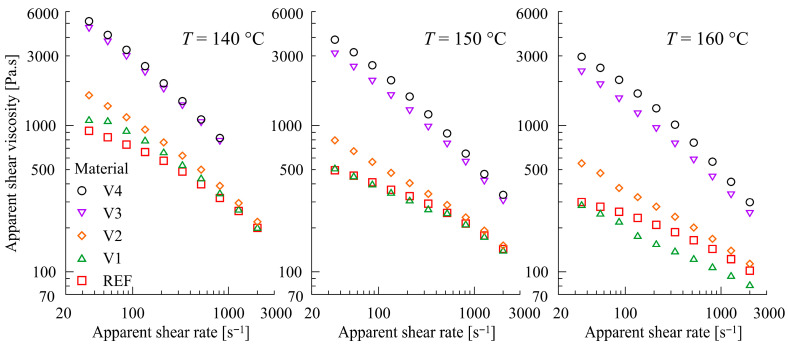
Effect of CECLs on shear deformation of REF and modified (V1 to V4) PBAT/PLA blends (arranged with respect to the individual temperatures).

Rheological properties are key to process optimization in film-blown extrusion. As can be seen in Figure 4, representing the shear viscosity data from the capillary rheometer for REF and modified PBAT/PLA blends (V1 to V4) at temperatures ranging from 140 to 160 °C, all blends exhibited a pseudoplastic behavior in the shear rate region relevant for extrusion. The addition of the CECLs increased the viscosity in the order (REF~V1) < V2 < V3 < V4, and the differences were most pronounced at the 160 °C. For the mutual positions of the REF and V1 subjects relative to the applied temperatures, see Figure 5. The results obtained for the modified blends correspond well with their molecular mass determined using Gel Permeation Chromatography (GPC) by Azevedo et al. [73]. The results showed significant increases in the molecular masses of V3 and V4 due to cross-linking reactions, where the number-average molar mass (*M*_n_ (V3) = 75,000, *M*_n_ (V4) = 72,000) nearly doubled that of REF (*M*_n_ (REF) = 44,000) (meaning that a single CECL molecule joins two polymer chains on average), whereas V1 and V2 have more or less identical molecular masses to REF (*M*_n_ (V1) = 42,000, *M*_n_ (V2) = 46,000). This indicates that, in the cases of V3 and V4, the CECL molecules react chemically with the polymer chains, forming a link between the two macromolecules of PBAT on average. Furthermore, a certain amount of some CECL may remain unreacted in V1 and V2. This explains the lower viscosity values of V1 and V2 (compared to V3 and V4), and does not exclude the grafting reactions of CECLs to macromolecules, see Figure 5. It is not without interest that a power approximation of the impact of apparent shear viscosity *η*_app_ on the apparent shear rate *γ*_app_ for V3 and V4 is of the form

(1) $$\eta_{\mathrm{app}}\;=\;\mathrm{Const}\;\times \;\gamma_{\mathrm{app}}^{-0.57}$$

with the fixed exponent −0.57 for all three temperatures (140 °C, 150 °C, 160 °C), see Figure 4 (left). This also documents the better performance of V3 and V4 from a rheological viewpoint, in the given temperature range and in comparison with V1 and V2.

**Figure 6:**
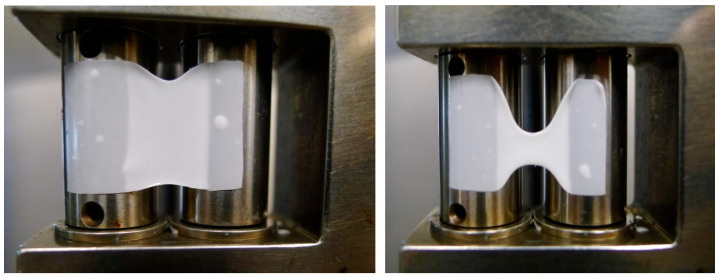
Deformation of REF rectangular samples: at 140 °C (**left**), and at 150 °C (**right**).

Prior to the measurement of elongational viscosities using an SER device, two principal conditions should be checked: the mild intensity of sample sagging during the process of temperature setting and an acceptably small deviation from the exponential decrease of sample cross-sections. For REF and V1–V4, both conditions are strongly violated, as shown in Figure 6. The impossibility of direct elongation measurements is also presented for the 80/20 wt% PLA/PBAT blend by Al-Itry et al. [14].

Other restrictions on the use of the SER Universal Testing Platform can be imposed if the sample does not rupture prior to the completion of the first revolution of the SER drums. However, this restriction can be ignored for thin samples with good adherence to the drums [82]. In this case, a sample does not have to overcome an abrupt increase in the drum diameter caused by clamps when starting the second revolution. It experiences only a negligible increase in diameter if the sample is adequately fixed at its ends (an increase from zero to the thickness of the lateral ends). In our case, the stretched film samples had to overcome only a gradual gentle slope, thereby increasing the drum diameter by approximately 2%.

Sagging behavior is governed by a sample’s temperature and the corresponding solid- and liquid-like behaviors. If the temperature interval between these two states is narrow, as in the case of PBAT/PLA compounds, the SER device can hardly be applied for measurements of elongational viscosities. This results from the fact that the measuring temperature should be established without temperature overshoots to maintain the morphological structure of the films. This requires long temperature setting times (with the final temperature achieved by a series of temperature settings from below) that may lead to unacceptable sagging. Moreover, sagging limits the application of SER devices and overhangs the upper film parts, leading to thickening along the upper film edge. The combination of these two effects prevents the proper determination of elongational viscosities. These phenomena diverge from the principal assumptions under which the SER device can be applied: exponentially decaying uniform sample thickness across the entire active narrowing rectangular area, see [74,75,76]. This behavior in the case of a PBAT/PLA blend is enhanced by the different melt temperatures of both components causing the simultaneous occurrence of solid- and liquid-like behavior of the individual immiscible components.

These shortcomings are interlaced with the term ’elongation at break’. Analogously, due to sagging, the precise values of this quantity cannot be determined; however, if we want to compare two materials—regardless of their geometrical form—using the SER device, the term ’elongation ratio at film rupture’ (*ER*_FR_) seems to be adequate and relates the time spans *t*_rupt_ that elapsed between the start of drum revolution with a fixed sample and the final film rupture for samples 1 and 2.

(2) $${ER}_{FR}=\frac{t_{rupt,1}}{t_{rupt,2}}$$

First, it was necessary to determine the temperatures at which the SER device could be used for the five PBAT/PLA blends investigated. Based on the range between 135 °C and 170 °C, it was found that the optimal common temperature interval is 150 ± 5 °C for the CECL-modified blends V1 to V4; for the material V4, this was found at even higher temperatures. In this interval, all PBAT/PLA blends exhibited optimally balanced fluid-like behaviors with acceptable sagging. Nevertheless, the remaining deviations of individual optimal temperatures may slightly worsen the comparisons among sample characterizations using *ER*_FR_ a little bit. Only in the case of REF, did the temperature have to be lowered to 140 °C, owing to intensive sagging at 150 °C (with the absence of chain-extending cross-linkers, the lower melt temperature of PBAT is more pronounced).

The thermo-mechanical properties of the blown films of REF, and V1 to V4 are anisotropic [71] because of the orientation effects of dispersed droplets in the melt [54] and polymer chains, both of which were introduced during blown film extrusion [36]. Ai et al. [40] showed that the tear strengths in the transverse direction (TD) always exceeded the tear strengths in the extrusion direction (ED).

**Figure 7:**
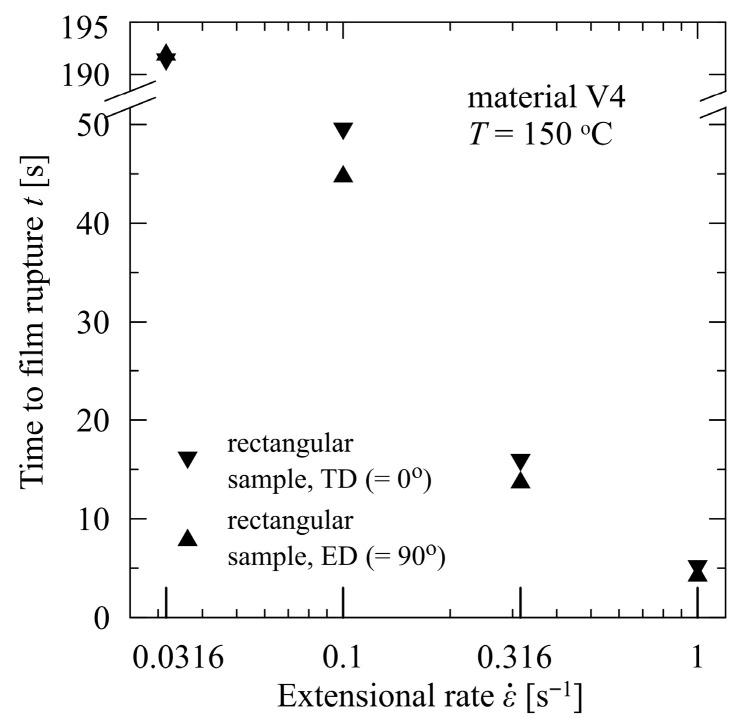
Times to film rupture of V4—PBAT/PLA, modified with poly (4,4-dicyclohexylmethane carbodiimide), in transverse and extrusion directions.

The differences of mean times to film rupture between the TD and the ED were less than 5% for each extensional rate, and the TD values always exceeded those in the ED (see Figure 7), showing that morphological formation differs with respect to the ED and TD during the blown film process. Not surprisingly, the time values in both directions for the lowest extensional rate (0.0316 s^−1^) are practically identical (differs by 0.5%); as such, for low extensional rates and a temperature of 150 °C, the process of disentanglement effectively contributes to morphological structural change. At these rates, the macromolecules have sufficient time to slip one out of the other, and the initial differences generated by the anisotropic film blowing conditions vanish completely. The values for a slant angle of 45° were also measured and are located between the two limiting (0° and 90°) values, as depicted in Figure 7 (for the sake of brevity, lack of clarity is not presented).

**Figure 8:**
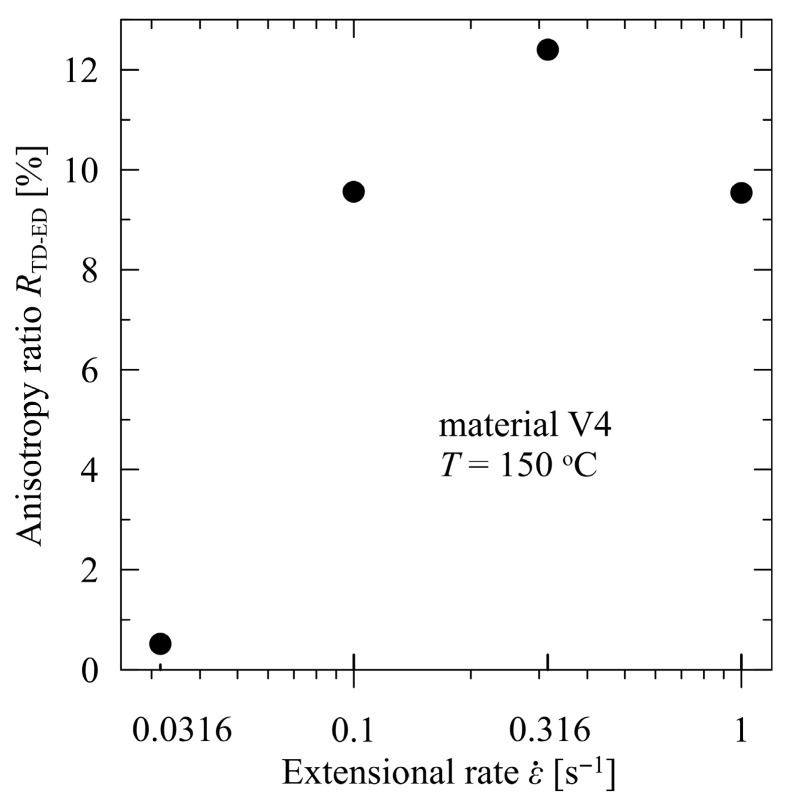
Anisotropy ratio of times to film rupture for V4—PBAT/PLA, modified with poly(4,4-dicyclohexylmethane carbodiimide), in extrusion and transverse directions.

The differences for other rates expressed by the anisotropy ratio *R*_TD-ED_

(3) $$R_{TD-ED}=\frac{t_{rupt,TD}-t_{rupt,ED}}{t_{rupt,TD}}100\%$$

attain approximately 10%, see Figure 8. From the viewpoint of the practical application of the studied materials (packaging material), the film rupture with respect to the elongated direction is relatively balanced with no dominating direction. This enables one to apply the studied material regardless of its orientation.

**Figure 9:**
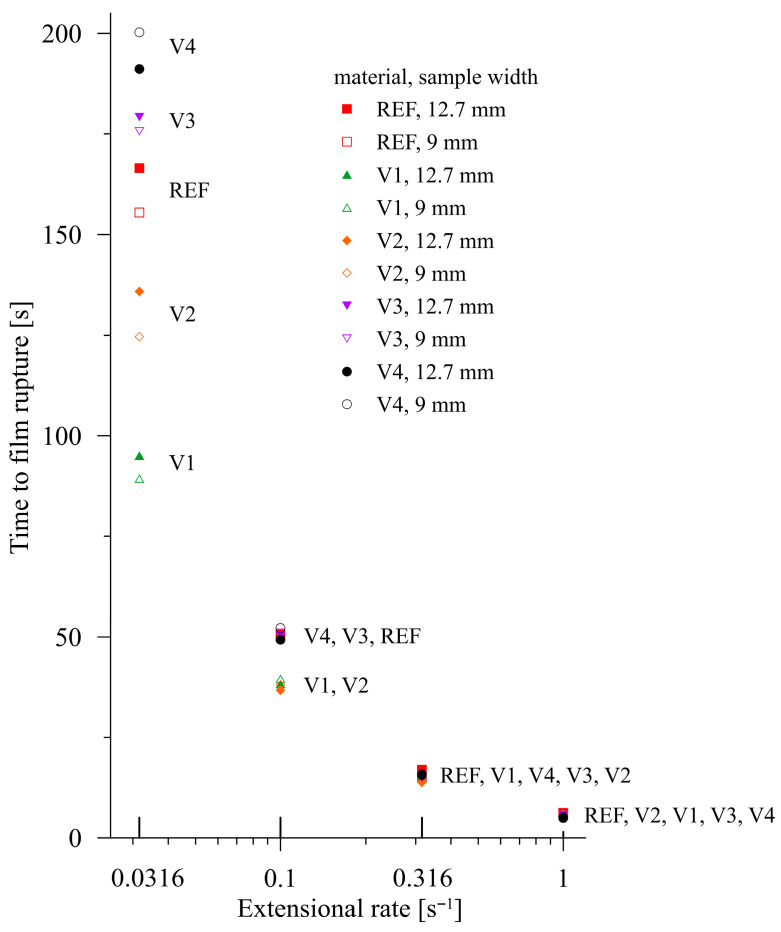
Elongational rate-dependent times to film rupture in the transverse direction. The experimental data are described according to the corresponding materials.

For all five materials, the time interval that elapsed between the start of the SER drum revolution and material rupture was measured. All measurements were performed for two widths of rectangular samples (transverse direction, 0°): 12.7 mm (maximum possible width in the SER device) and 9 mm. Each measurement was conducted at least in triplicate and the results are shown in Figure 9. The measurements of V1–V4 were carried out at 150 °C, while for REF, it was at 140 °C due to the intensive sagging of REF at higher temperatures, as stated above. This means that the data for REF introduced in Figure 9 are only of an informative character and correspond to the highest temperature under which an extensional measurement can be carried out.

**Figure 10:**
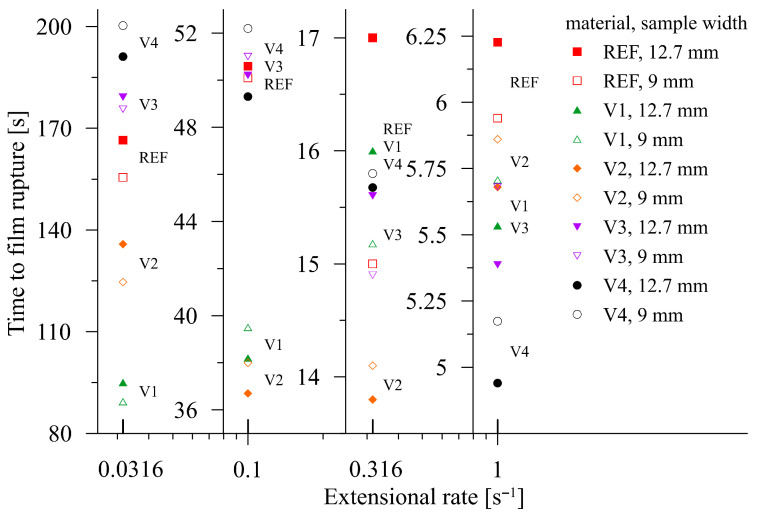
Elongational rate-dependent times to film rupture in the TD, individual charts. The experimental data are described according to the corresponding materials.

For improved legibility, Figure 9 was converted from a global view to a version localized according to the individual extensional rates, as shown in Figure 10.

It is apparent that the initial macromolecular interplay at low extension rates favors disentanglement. It is replaced by consecutive passages to strain hardening, where the rupture time intervals for the pure PBAT/PLA blend significantly dominate. It is necessary to remember that the reference PBAT/PLA blend (denoted as REF) was measured at 140 °C, i.e., at 10 °C lower than the modified samples V1–V4. This difference is suppressed for lower extension rates (as for 0.0316 s^−1^) due to the process of disentanglement, as discussed above. The dominance of this process is documented by intermixing REF values with the modified samples, regardless of the temperature under which the experiments were carried out.

With increasing extensional rates, strain hardening begins to compete and successively replaces the process of disentanglement. This can be demonstrated for REF at an extensional rate of 1 s^−1^ at 140 °C, with REF having the highest values of time to film rupture. However, at 150 °C, REF exhibits a pronounced liquid-like behavior, preventing elongation measurements. This is caused by the absence of chain-extending cross-linkers and the dominating position of PBAT (PBAT/PLA = 6:1), which exhibits a lower melt temperature. In contrast, decreasing the temperature by 10 °C reduces the liquid-like behavior of the modified samples, which is reflected in the apparent prolongation of breakage time (for instance, by 17% for V1, with a sample width of 12.7 mm, and an extensional rate of 1.0 s^−1^).

**Figure 11:**
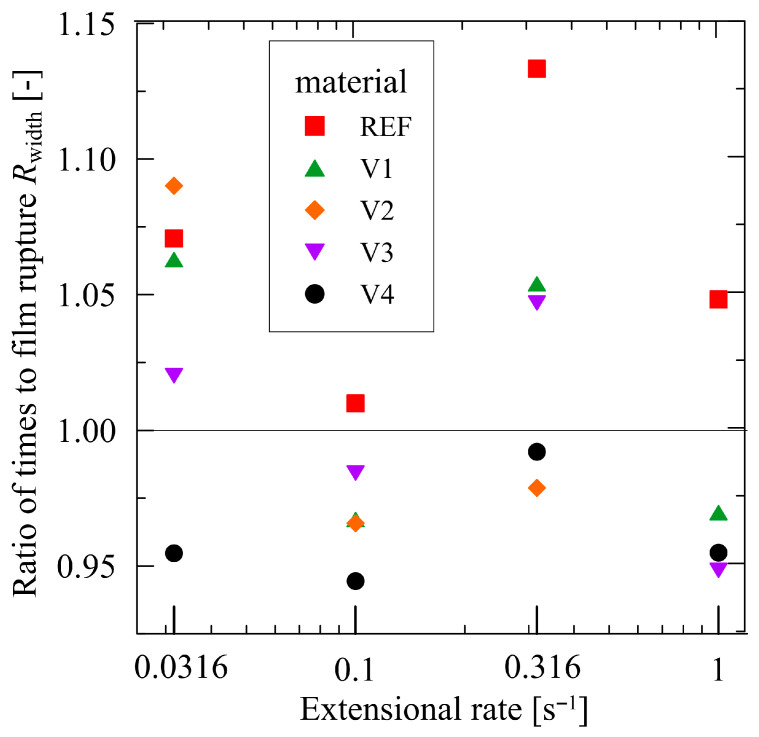
Ratios of times to film rupture of width 12.7 mm to width 9 mm.

The practical coincidence of the results is expressed through a ratio relating the times to film fracture for both widths

(4) $$R_{width}=\frac{t_{rupt,12.7\;mm}}{t_{rupt,9\;mm}}$$

which documents the excellent homogeneity of the prepared materials without air bubbles and with an initial uniform thickness due to good mixing during melt compounding. The dispersion of the ratio *R*_width_ is limited to the interval between 0.95 and 1.1, as shown in Figure 11, which corresponds to the experimental errors.

The obtained results justify the introduction of the quantity ‘elongation ratio at the film rupture *ER*_FR_, see rel. (2), by comparing the elongational attributes of the modified blends. For the interpretation of the measurements using the SER device, one has to keep in mind that V1 and V2 may contain the unreacted rests of their CECLs, easing the chain slipping of macromolecules owing to their softening effects, and that V3 and V4 consist of longer macromolecules; thus, they have higher entanglement or cross-linking densities with a more pronounced strain hardening behavior, which is crucial for blown film extrusion. For instance, when comparing V4 and REF materials, the *ER*_FR_ value attains 0.8 for $\dot{\varepsilon }$ = 1.0 s^−1^, proving an apparent contribution of the CECL 4 (poly (4,4-dicyclohexylmethane carbodiimide)) addition. This chain-extending cross-linker exhibits the best characteristics for improving the blown film process. As presented above, the materials V3 and V4 are in many respects comparable; however, the better performance of V4 from a practical viewpoint is given by the fact that the used CECL (poly (4,4-dicyclohexylmethane carbodiimide)) contributes to the better compactness of two seemingly inconsistent basic components: PBAT and PLA. As documented by SEM characterization (Figure 3), better interface adhesion is exhibited by V4. The two components (PBAT and PLA) exhibit different—not overlapping—ranges of melt viscosities. It was verified that V3 cannot exceed 160 °C in its processing due to its full liquid-like behavior in contrast to V4, which can be processed even at 170 °C (the corresponding data were not included in the figures), and which displays more suitable strain hardening behavior. This means that production rate of V4 can be accelerated due to its viscosity reduction at higher temperatures, reflected in its lower flow resistance. Another positive input is the better invariantness of V4 in comparison to V3, which concerns its orientation (see Figure 11) as the characteristics (as a packaging material) are nearly direction-invariant.

## 4. Conclusions

In the process of blown film extrusion, both shear and elongational behaviors represent very important characteristics. Shear viscosity data showed the well-distinguished effect of chain-extending cross-linkers on the flowability of PBAT/PLA blends, reflected in their molecular weights. An elongational viscosity measurement is rather limited with the SER Universal Testing Platform because of the considerably higher sensitivity of PBAT/PLA blends to temperature changes, resulting in a fast transition to fluidity. However, the SER was found to provide a very good performance when two (or more) materials were compared. It was shown that for very low extensional rates, the elongational process is dominated by the disentanglement of the studied materials, preventing strain hardening. Material responses to both shear and elongational deformation confirmed that out of the four chain-extending cross-linkers, the best contribution to blown film processing was exhibited by poly (4,4-dicyclohexylmethane carbodiimide), as it improved PBAT/PLA interfacial adhesion, enabling processing at higher temperatures with better strain hardening and lower flow resistance, and offers faster production rates and nearly direction-independent packaging properties.

## Figures and Tables

**Table 1:** Mean thicknesses and draw ratios of films of REF and V1 to V4.

Material	Thickness in µm	Draw Ratio
REF	98.8 ± 1.3	3.24
V1	101.0 ± 1.6	3.17
V2	100.2 ± 1.3	3.19
V3	100.4 ± 1.7	3.19
V4	101.4 ± 1.7	3.16

## Data Availability

The data supporting the findings of this study are available from the corresponding author upon request.
